# Frequency, Management, and Outcomes of Outpatient Hyperkalemia: A Population-Based Cohort Study

**DOI:** 10.1177/20543581251356568

**Published:** 2025-07-29

**Authors:** Michael Chiu, Nivethika Jeyakumar, Graham Smith, Danielle M. Nash, Mohamed Abou El Hassan, Dana Bailey, Peter Catomaris, Kika Veljkovic, Louise Moist, Amit X. Garg, Arsh K. Jain

**Affiliations:** 1 Division of Nephrology, Department of Medicine, Western University, and London Health Sciences Centre, ON, Canada; 2 Kidney Clinical Research Unit, London Health Sciences Centre, ON, Canada; 3 ICES Western, London Health Sciences Centre Research Institute, ON, Canada; 4 Department of Epidemiology and Biostatistics, Western University, London, ON, Canada; 5 Department of Laboratory Medicine and Molecular Diagnostics, Sunnybrook Health Sciences Centre, University of Toronto, ON, Canada; 6 Department of Laboratory Medicine and Pathobiology, University of Toronto, ON, Canada; 7 LifeLabs Medical Laboratory Services, Toronto, ON, Canada; 8 Department of Pathology, Dalhousie University, Halifax, NS, Canada; 9 Department of Pathobiology and Laboratory Medicine, Sinai Health, University of Toronto, ON, Canada; 10 Dynacare, Brampton, ON, Canada; 11 LifeLabs, Toronto, ON, Canada

**Keywords:** hyperkalemia, chronic kidney disease, laboratory reporting

## Abstract

**Background:**

Hyperkalemia is a potentially life-threatening condition, with guidelines recommending urgent treatment when the serum potassium level is greater than 6.0 mmol/L. However, these recommendations are inconsistent, leading to diverse approaches to patient care.

**Objectives:**

The primary objectives were to use population-based datasets to determine how often outpatient hyperkalemia (K > 6.2 mmol/L) occurs and how frequently patients present to the emergency department (ED) within 24 hours of the hyperkalemia report. Secondary objectives were to compare the characteristics of patients who had an ED encounter to those who did not, assess clinical outcomes within 7 days of the hyperkalemia report, and describe the initial potassium result within 24 hours of an ED encounter.

**Design:**

Retrospective cohort study using linked population-based datasets at ICES.

**Setting:**

Ontario, Canada from January 1, 2007, to December 24, 2021.

**Patients:**

Adult patients (≥18 years) not on dialysis with an outpatient hyperkalemia result >6.2 mmol/L who were identified through flagged and urgently communicated results from outpatient laboratories.

**Measurements:**

Emergency department encounters within 24 hours following an outpatient serum potassium report >6.2 mmol/L. Outcomes included all-cause mortality, cardiovascular mortality, arrhythmias, cardiac arrest in the ED, hospitalizations, and new dialysis starts within 7 days of the hyperkalemia report.

**Methods:**

Administrative healthcare data were linked with laboratory results to compare baseline characteristics, medication use, healthcare utilization, and clinical outcomes for all patients. Standardized differences were used for comparisons.

**Results:**

There were over 65 million serum potassium measurements and 57 607 individuals with an outpatient hyperkalemia value >6.2 mmol/L. Of these, 7469 (13.0%) individuals had an ED encounter within 24 hours. Individuals with an ED encounter had more comorbidities, higher medication use, and more prior healthcare utilization. Within 7 days of the hyperkalemia report, 675 of the 57 607 individuals (1.2%) had died. Where data were available, the first potassium value within 24 hours of an ED encounter was 1.5 mmol/L (± SD 1.3) lower, on average, than the initial outpatient potassium value.

**Limitations:**

All-cause mortality may not be attributable to the hyperkalemia result. Sudden cardiac death, which is more specific to hyperkalemia, is not completely captured in our data sources. Data for medications are limited to patients 65 years of age and older.

**Conclusions:**

Outpatient hyperkalemia is common. Despite guidelines recommending urgent treatment for patients with serum potassium levels >6.2 mmol/L, most are not referred to the ED.

## Abrégé

**Contexte:**

L’hyperkaliémie est une affection potentiellement mortelle. Les lignes directrices recommandant un traitement urgent lorsque le taux de potassium sérique est supérieur à 6,0 mmol/l. Ces recommandations ne sont toutefois pas appliquées de façon uniforme, ce qui se traduit par des approches diverses pour les soins aux patients.

**Objectifs:**

L’objectif principal était d’utiliser des ensembles de données populationnelles pour déterminer la fréquence de l’hyperkaliémie (K > 6,2 mmol/l) mesurée en consultation externe et la fréquence des visites aux urgences dans les 24 heures suivant un rapport d’hyperkaliémie. Les objectifs secondaires étaient la comparaison des caractéristiques des patients selon qu’ils avaient ou non visité l’urgence, l’évaluation des issues cliniques dans les 7 jours suivant un rapport d’hyperkaliémie et la description du taux initial de potassium dans les 24 heures suivant une visite aux urgences.

**Conception:**

Étude de cohorte rétrospective menée à partir des données de santé couplées de l’ICES pour la population étudiée.

**Cadre:**

Ontario (Canada) du 1^er^ janvier 2007 au 24 décembre 2021.

**Sujets:**

Des adultes (≥18 ans) non dialysés avec une hyperkaliémie (K > 6,2 mmol/l) mesurée en ambulatoire, identifiés grâce à des résultats signalés et communiqués d’urgence par des laboratoires externes.

**Mesures:**

Les visites aux urgences dans les 24 heures suivant le rapport en consultation externe d’un taux de potassium sérique supérieur à 6,2 mmol/l. Les issue cliniques comprenaient la mortalité toutes causes confondues, la mortalité cardiovasculaire, les arythmies, un arrêt cardiaque au service des urgences, les hospitalisations et l’initiation de la dialyse dans les 7 jours suivant le rapport d’hyperkaliémie.

**Méthodologie:**

Les données administratives de santé ont été couplées aux résultats de laboratoire afin de comparer les caractéristiques initiales, l’utilisation des médicaments, l’utilisation des soins de santé et les issues cliniques de tous les patients. Les différences normalisées ont été utilisées pour les comparaisons.

**Résultats:**

Plus de 65 millions de mesures de potassium sérique ont été répertoriées. En tout, 57 607 individus avaient eu un rapport d’hyperkaliémie (K > 6,2 mmol/l) en consultation externe. De ce nombre, 7469 (13,0 %) personnes avaient visité les urgences dans les 24 heures, et ces personnes avaient davantage de comorbidités, prenaient plus de médicaments et avaient plus souvent utilisé les soins de santé. Des 57 607 personnes identifiées, 675 (1,2 %) étaient décédées dans les 7 jours suivant le rapport d’hyperkaliémie. Lorsque les données étaient disponibles, la valeur initiale de potassium dans les 24 heures suivant la visite aux urgences était réduite de 1,5 mmol/l (ÉT±1,3) en moyenne par rapport à la valeur initiale mesurée en consultation externe.

**Limites:**

La mortalité toutes causes confondues pourrait ne pas être attribuable à l’hyperkaliémie. La mort subite de cause cardiaque, qui est plus spécifique à l’hyperkaliémie, n’était pas complètement saisie dans nos sources de données. Les données sur les médicaments étaient limitées aux patients âgés de 65 ans et plus.

**Conclusion:**

L’hyperkaliémie est fréquemment détectée en consultation externe. Bien que les lignes directrices recommandant le traitement urgent des patients présentant un taux de potassium sérique supérieur à 6,2 mmol/l, la plupart de ceux-ci ne sont pas orientés vers le service des urgences.

## Introduction

Outpatient hyperkalemia is common and has potentially life-threatening consequences, yet its management remains inconsistent and controversial.^1-3^

Several factors contribute to this controversy. First, the accuracy of potassium measurements can be compromised,^4^ as several factors can lead to a pre-analytical error resulting in an elevated potassium value (ie, factitious hyperkalemia).^5-8^ Second, the reporting guidelines used by community laboratories to alert healthcare providers of hyperkalemia results are inconsistent.^9,10^ Finally, the lack of evidence in defining “severe” hyperkalemia, has resulted in guidelines with different thresholds for urgent treatment in a hospital setting (ie, requiring emergency department [ED] encounter).^9^ The guidelines have been largely informed by a diagnosis of hyperkalemia from inpatient and ED settings, which may not be applicable to outpatient management (Supplemental Appendix A).^9^ Specifically, the Canadian Cardiovascular Society and KDIGO recommend urgent treatment of hyperkalemia at a threshold of >6.0 mmol/L whereas the Renal Association recommends urgent treatment with a threshold of >6.5 mmol/L.^11-16^

Amongst all the uncertainty, it also remains unclear how healthcare providers act after receiving a hyperkalemia report (ie, send patient to the ED versus outpatient treatment). Healthcare providers must determine whether to initiate outpatient treatment, repeat the potassium measurement, or urgently refer the patient to the ED. Better information on practice patterns and outcomes would inform future guidelines and clinical practice.

The primary objectives of this study were to (1) determine how frequently hyperkalemia (K >6.2 mmol/L) occurs in the outpatient setting; and (2) determine how often patients have an ED encounter within 24 hours after the potassium result is reported to the provider. The secondary objectives of this study were (1) for outpatients with hyperkalemia, to compare those who had an ED encounter within 24 hours of the report versus those who did not; (2) for outpatients with hyperkalemia, to determine outcomes within 7 days of the hyperkalemic report; and (3) for outpatients who present to the ED within 24 hours of their hyperkalemic report, to compare the outpatient potassium result with the initial ED potassium result.

## Methods

### Study Design and Population

This was a retrospective population-based cohort study using the linked administrative healthcare databases held at ICES for Ontario, Canada. Ontario, with a population of over 15 million residents, has a single-payer universal healthcare system.^17^ The healthcare system encompasses all physician services, ambulatory care, in-hospital care, and outpatient laboratory services. In addition, everyone 65 years of age and older has access to drug coverage through the Ontario Drug Benefit (ODB) program. Information on resident use of healthcare services is collected and linked using unique encoded identifiers at ICES. ICES is an independent, nonprofit research institute whose legal status under Ontario’s health information privacy law allows it to collect and analyze healthcare and demographic data, without consent, for health system evaluation and improvement. The use of data in this project was authorized under section 45 of Ontario’s Personal Health Information Protection Act, which does not require review by a Research Ethics Board. The conduct and reporting of the study followed the Reporting of Studies Conducted Using Observational Routinely Collected Health Data (RECORD) guidelines for observational studies (Supplemental Appendix B).^18^

All potassium results above 5.0 mmol/L were identified. Cases of mild hyperkalemia, defined as a potassium value of greater than 5.5 mmol/L were also identified. For the purposes of this study, hyperkalemia was defined as a serum potassium exceeding 6.2 mmol/L, which is the threshold recommended by the Ontario Association of Medical Laboratories for alerting health providers. Laboratories are also required to contact providers immediately and directly for values exceeding 6.6 mmol/L.^10^ Data from the Ontario Laboratory Information System (OLIS) provide multiple lab processing timepoints. Important lab processing timepoints that are reported on OLIS (ie, potassium collection and reporting) were determined in consultation with the clinical biochemists from the main outpatient laboratories. The outpatient laboratories verified that the timepoints for when the potassium was collected and reported were accurate.

In this study, all adult patients ≥18 years with a potassium result >6.2 mmol/L between January 1, 2007 and December 24, 2021 were eligible for inclusion. The first eligible value was used for patients with multiple hyperkalemic values. The index date was defined as an ED visit within 24 hours of when the outpatient hyperkalemia result was reported by the community laboratory. Patients were excluded if (1) the potassium reporting time was >48 hours, as specimens with delayed reporting are suggestive of processing errors; (2) they were receiving chronic dialysis; (3) the initial lab value was obtained in the ED (between a hospital or ED admission and discharge); (4) the lab value was reported between a hospital or ED admission and discharge (as a provider would not have had an opportunity to act upon the report as the patient was already in a hospital setting); (5) there was a hospital or ED encounter prior to when the potassium result was reported (patient more likely to have presented to the ED for a different reason); (6) the patient died on the date of the hyperkalemia report (as there is no way to act on the report); and (7) the patient did not have a baseline serum creatinine value as potassium is usually interpreted in the context of kidney function.

### Baseline Characteristics and Data Sources

We used linked health administrative databases at ICES to collect patient information. Patients were characterized by their demographics (age, sex, and rurality), healthcare utilization in the past year (hospital encounters, ED encounters, family physician encounters, and nephrologist encounters), comorbidities, laboratory values, and prescription medication use.

The following linked health administrative databases housed at ICES were used in this study. The *Registered Persons Database* (*RPDB*) contains information on patient demographics, including sex, birth, and death dates. *National Ambulatory Care Reporting System* (*NACRS*) provided data for community and hospital-based ambulatory care encounters (ED encounters). The *Ontario Health Insurance Plan* (*OHIP*) database contains records of all physician claims for services, including service date, procedures performed, and associated diagnoses. The *Canadian Institute for Health Information Discharge Abstract and Same Day Surgery Databases* (*CIHI-DAD and CIHI-SDS*) capture all diagnostic and procedural information for all hospitalizations. The *Canadian Organ Replacement Registry* (*CORR*) contains information on patients receiving maintenance dialysis. The *Ontario Diabetes Database* (*ODD*) identifies patients with diabetes. The *ICES Physician Database* (*IPDB*) provides information about all physicians who have practiced in Ontario and was used to identify the specialty of physicians who ordered the laboratory tests. *OLIS* provides access to lab results from all community and most hospital laboratories in the province. This provided information on blood test characteristics and test results. The *Ontario Drug Benefit* (*ODB*) database provides information on publicly funded outpatient drug prescriptions for individuals over the age of 65 or people with special considerations receiving drug benefits. The *Office of the Registrar General Database* (*ORGD*) provides information on the cause of death.

### Clinical Outcomes

The main clinical outcome of interest was all-cause mortality. Additional clinical outcomes were cardiovascular mortality, arrhythmias, performing cardiac resuscitation in the ED, hospitalizations, and dialysis starts. To determine the immediate and short-term consequences of outpatient hyperkalemia, outcomes were reported at 1, 3, and 7 calendar days after the potassium report. Subgroup analyses were performed for all-cause mortality based on diabetes diagnosis, level of chronic kidney disease (eGFR ≥ 30 and eGFR <30), and prescriptions for renin-angiotensin aldosterone inhibition (RAASi). This was defined as prescription for angiotensin-converting enzyme inhibitors (ACEi), angiotensin receptor blockers (ARB), and spironolactone for patients who were ODB-eligible.

### Sensitivity Analysis

The cohort was rebuilt without exclusions (4) and (5), which included cases when the lab value was reported between a hospital or ED admission and discharge and when there was a hospital or ED encounter before the potassium level was reported. It is possible that these patients presented to the ED for symptomatic hyperkalemia, such as muscle weakness or palpitations. All analyses were repeated for this cohort.

A sensitivity analysis was also conducted to assess the difference in rates of adverse outcomes for a higher severity of hyperkalemia. All-cause mortality was assessed for potassium values exceeding 6.5 mmol/L, which is in line with guidelines suggesting urgent treatment of hyperkalemia.

### Initial ED Potassium Result

Not all hospitals reported laboratory values to OLIS within the study time frame. Therefore, the initial ED potassium result was restricted to patients who, at the time of the blood measurement, presented to ED while their primary home residence was within the geographical catchment area of a hospital that was actively contributing laboratory results to OLIS.^19^ The potassium value within 24 hours following the ED registration date was used as the initial ED potassium result.

### Statistical Analysis

Baseline characteristics for patients who presented to the ED and those who did not were summarized using descriptive statistics. Continuous measures were expressed as means (standard deviation, SD) or medians (interquartile range, IQR), and categorical measures were expressed as frequency (proportion, %). A standardized difference >10% was considered a meaningful difference between groups.^20^ The proportion of patients with a hyperkalemic event meeting each outcome of interest was calculated. All analyses were conducted using SAS version 9.4 (SAS Institute, Cary, North Carolina).

## Results

### Frequency of Outpatient Hyperkalemia and Subsequent ED Encounters

**Figure 1.**
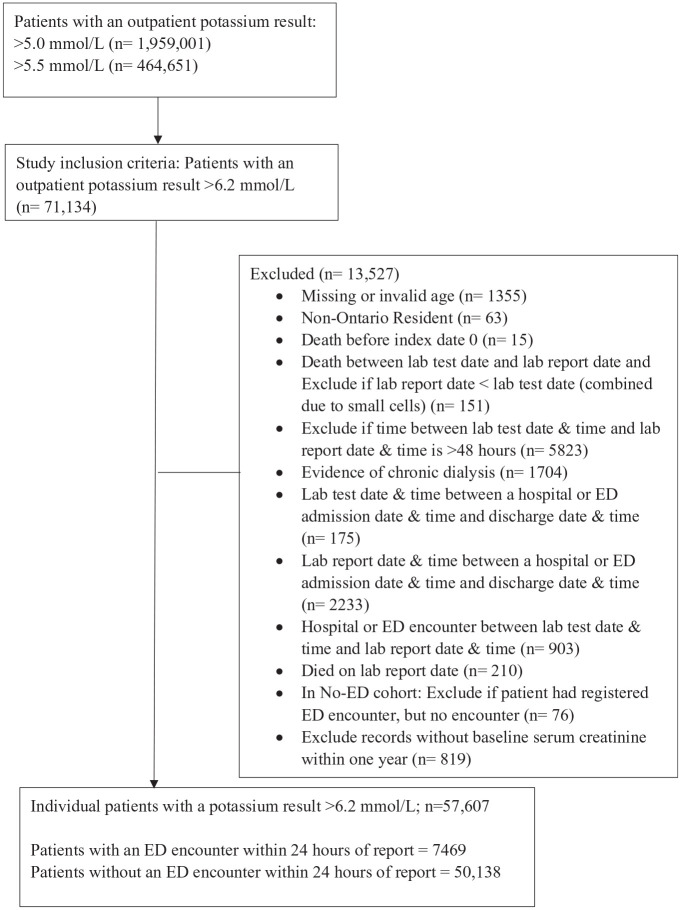
Patient Population.

Figure 1 describes the patient population. Between January 1, 2007 and December 24, 2021, there were 1 959 001 patients with hyperkalemia (potassium value >5.0 mmol/L). Specifically, there were 71 134 patients with a potassium value >6.2 mmol/L. After data cleaning steps and exclusions were applied, there were a total of 57 607 individual patients with a potassium value >6.2 mmol/L. Of these patients, 7469 (13.0%) had an ED encounter within 24 hours of the potassium report and 50 138 patients did not have such an encounter (No-ED).

### Comparison of Patients with an ED Encounter Versus No-ED Encounter

#### Demographics

Patients with an ED encounter within 24 hours and those without an ED encounter within 24 hours of the potassium report are selectively described and compared in Table 1 (see Supplemental Appendix C for full Table 1). In the entire cohort, the mean age was 65 (SD ± 18) years and 48% were women. Most people (91%) lived in an urban setting.

**Table 1.** Baseline Characteristics of Patients With an Outpatient Hyperkalemic Result.

	Full population	ED encounter	No-ED encounter	Std. Diff^a^
	**57,607**	**7469**	**50,138**	
**Characteristic**	**N (%)**	**N (%)**	**N (%)**	
*Demographics*				
Age, years, Mean (SD)	64.9 (18.2)	69.8 (15.4)	64.2 (18.4)	33%
Female	27,503 (47.7)	3389 (45.4)	24,114 (48.1)	5%
Rurality^b^	5081 (8.8)	827 (11.1)	4254 (8.5)	9%
*Health care utilization in the prior year*				
Hospital visits, Mean (SD)	0.6 (1.4)	1.1 (1.9)	0.6 (1.8)	
ED visits, Mean (SD)	1.0 (2.0)	1.6 (2.7)	0.9 (1.8)	
Family physician visits, Mean (SD)	11.3 (12.7)	13.0 (14.5)	11.1 (12.4)	
Nephrologist visits, Mean (SD)	0.7 (2.5)	1.2 (3.5)	0.6 (2.3)	
*Comorbidities, %*				
Charlson score, Mean (SD)	0.8 (1.6)	1.4 (2.0)	0.8 (1.6)	35%
Diabetes	26,723 (46.4)	4360 (58.4)	22,363 (44.6)	28%
Congestive Heart Failure	10,886 (18.9)	2108 (28.2)	8778 (17.5)	26%
Hypertension	38,470 (66.8)	5893 (78.9)	32,577 (65.0)	31%
Kidney transplant	234 (0.4)	52 (0.7)	182 (0.4)	4%
*Laboratory Measurements in the prior year*				
Index potassium value, Mean (SD)	6.8 (0.78)	6.8 (0.65)	6.8 (0.80)	3%
eGFR (ml/min/1.73 m^2^), Mean (SD)	65.0 (33.5)	47.6 (30.2)	67.6 (33.2)	63%
Urine ACR (mg/mmol)				
Mean (SD)	48.63 (117)	67.61 (138)	45.1 (113)	18%
Median (IQR)	4 (1-30)	10 (2-64)	3 (1-25)	
Missing	6223 (10.8)	3564 (47.7)	29,147 (58.1)	21%
*Medication use in the prior 4 months, for those eligible for ODB, %*				
ODB eligible	32,023 (55.6)	5078 (68.0)	26,945 (53.7)	30%
ACE inhibitor	12,837 (40.1)	2118 (41.7)	10,719 (39.8)	4%
Angiotensin receptor blocker	9547 (29.8)	1533 (30.2)	8014 (29.7)	1%
Antibiotics	10,818 (33.8)	1920 (37.8)	8898 (33.0)	10%
Aliskiren	178 (0.6)	25 (0.5)	153 (0.6)	1%
Beta blockers	13,681 (42.7)	2438 (48.0)	11,243 (41.7)	13%
Calcineurin inhibitors	219 (0.7)	49 (1.0)	170 (0.6)	4%
Loop diuretics	9272 (29.0)	1759 (34.6)	7513 (27.9)	14%
Potassium sparing diuretics	5253 (16.4)	1081 (21.3)	4172 (15.5)	15%
Thiazide diuretics	3866 (12.1)	659 (13.0)	3207 (11.9)	3%
NSAIDs	3891 (12.2)	525 (10.3)	3366 (12.5)	7%
Potassium supplements	750 (2.3)	111 (2.2)	639 (2.4)	1%
SGLT2 inhibitors	1227 (3.8)	220 (4.3)	1007 (3.7)	3%
Spironolactone	4692 (14.7)	988 (19.5)	3704 (13.7)	16%
Potassium binders	1844 (5.8)	402 (7.9)	1442 (5.4)	10%

*Note.* SD = standard deviation; IQR = interquartile range; ODB = Ontario drug benefit; OLIS = Ontario laboratory reporting system; eGFR = estimated glomerular filtration rate; ACR = albumin-to-creatinine ratio; ACE = angiotensin-converting enzyme; NSAID = nonsteroidal inflammatory drug; SGLT2 = sodium-glucose transporter 2.

a Standardized difference was used to compare hyperkalemic patients who presented to an ED versus hyperkalemic patients who did not present to an ED. Standardized differences are less sensitive to sample size than traditional hypothesis tests. They provide a measure of difference between groups with respect to a pooled standard deviation. A standardized difference ≥1% is considered a meaningful difference between groups.

b Rural status was defined as residence within a community <10 000 persons.

#### Health care utilization in the prior year

Compared with patients without an ED encounter, in the prior year the ED group had more ED encounters on average (1.6 vs 0.9) and more hospitalizations (1.1 vs 0.6). They also had more prior encounters with a family physician (13.0 vs 11.1) and a nephrologist (1.2 vs 0.6), on average.

#### Comorbidities

The Charlson Comorbidity Index was higher in the group with an ED encounter: 23.7% with a score of 3+ in the ED group versus 12.5% in the No-ED group. The ED group compared with the No-ED group, had a greater proportion of people with diabetes (58.4% vs 44.6%), hypertension (78.9% vs 65.0%), and congestive heart failure (28.2% vs 17.5%).

#### Baseline laboratory measurements

**Figure 2.**
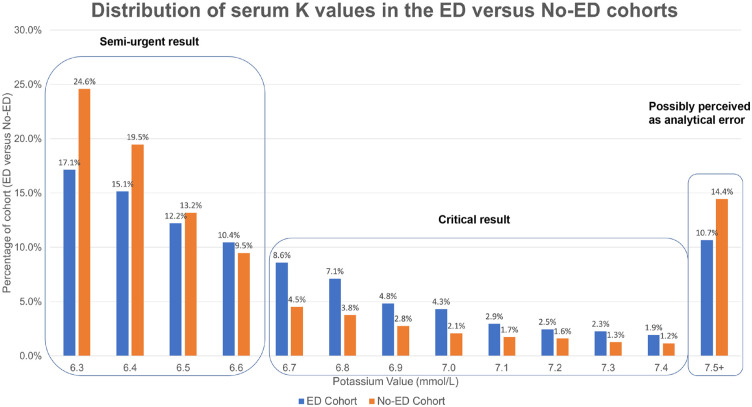
Distribution of potassium values in the ED and No-ED groups. *Note.* Values >9.0 are considered incompatible with life by community laboratories.

In the entire population, the mean outpatient potassium was 6.8 mmol/L (SD ± 0.8), with no difference between the 2 groups. The distribution of the potassium values followed a normal distribution, with lower frequencies at higher potassium concentrations (Figure 2). In addition, the proportion of patients with an ED encounter within 24 hours of the hyperkalemia report in the population was 11% for patients with potassium values between 6.3 and 6.6 mmol/L and increased to 21% for patients with values between 6.7 and 7.4 mmol/L. When the potassium result was >7.5 mmol/L, 10% of patients had an ED encounter within 24 hours.

The mean estimated glomerular filtration rate (eGFR) was lower in the ED group compared with the No-ED group (48 vs 68 mL/min/1.73 m^2^). The mean urine albumin-to-creatinine ratio (ACR) was higher in the ED group compared with the No-ED group (68 vs 45 mg/mmol).

#### Baseline medication use

In the full cohort for those eligible for ODB coverage, 40% of patients had been prescribed ACEi and 30% were on ARBs in the 4 months prior to the study. There was no difference in their use between the 2 groups. There were more patients in the ED cohort prescribed β-blockers compared with the No-ED cohort (48.0% vs 41.7%), loop diuretics (34.6% vs 27.9%), and potassium sparing diuretics (21.3% vs 15.5%). Specifically, spironolactone was more commonly prescribed in the ED cohort (19.5% vs 13.7%). Potassium binders were prescribed more often in the ED cohort compared with the No-ED cohort (7.9% vs 5.4%). There was no significant difference in the use of thiazide diuretics, prescription nonsteroidal anti-inflammatory drugs, sodium-glucose transport protein 2 inhibitors (SGLT2i), or aliskiren.

### Aggregate Outcomes

The aggregate outcomes of this study are summarized in Table 2. For all patients with a potassium result >6.2 mmol/L, there were a total of 675 deaths (1.2% of the population) within 7 days of the hyperkalemia report. Among these deaths, 147 (0.3% of the population) occurred within 1 calendar day of the report and 382 of these deaths (0.7% of the population) occurred within 3 calendar days of the report.

**Table 2.** Aggregate Outcomes in Full Population (N = 57,607).

	Potassium report + 1 calendar day	Potassium report + 2 calendar days	Potassium report + 3 calendar days	Potassium report + 7 calendar days
	**N (%)**	**N (%)**	**N (%)**	**N (%)**
*Primary Outcomes*				
All-cause mortality	147 (0.3)	N/A	382 (0.7)	675 (1.2)
*Secondary Outcomes*				
Cardiovascular mortality	39 (0.1)	N/A	93 (0.2)	155 (0.4)
Arrhythmia	368 (0.6)	N/A	471 (0.8)	579 (1.0)
Running a cardiac arrest	1281 (2.2)	N/A	1546 (2.7)	1782 (3.1)
All-cause hospitalizations	2174 (3.8)	N/A	2837 (4.9)	3609 (6.3)
Any dialysis	141 (0.2)	N/A	198 (0.3)	260 (0.5)
*Descriptive Outcomes*				
Electrocardiogram test	8051 (14.0)	N/A	9716 (16.9)	11,527 (19.5)
Total potassium binder prescriptions	4059 (7.0)	N/A	5096 (8.8)	6060 (10.5)
New potassium binder prescriptions^a^	3719 (91.6)	N/A	4655 (91.3)	5494 (90.7)
Repeat serum potassium test	7983 (13.9)	12,227 (21.2)	15,245 (26.5)	25,479 (44.2)

a New potassium binder prescriptions are a proportion of the total potassium binder prescriptions.

Subgroup analyses are summarized in Table 3. In the full population, there were 11 322 patients with an eGFR <30 mL/min/1.73 m^2^. Of these patients, there were a total of 461 deaths (4.1%) in 7 days. Of these deaths, 124 (1.1%) occurred within 1 calendar day of the report and 276 (2.4%) occurred within 3 calendar days. In the population, there were 32 023 patients who were ODB-eligible. Among those ODB-eligible, there were 22 814 patients on RAASi versus 9209 patients not on RAASi. For patients on RAASi, there were a total of 358 deaths (1.6%) at 7 days, where 91 (0.4%) occurred at 1 calendar day and 212 (0.9%) occurred at 3 calendar days. In the patients without RAASi, there were 233 (2.5%) deaths at 7 days, where 58 (0.6%) occurred at 1 calendar day and 131 (1.4%) occurred at 3 calendar days.

**Table 3.** Subgroup Analyses of All-Cause Mortality in the Full Population (N = 57,607).

		Potassium report + 1 calendar day	Potassium report + 3 calendar days	Potassium report + 7 calendar days
	Total N	**N (%)**	**N (%)**	**N (%)**
Diabetes status				
Yes	26,723	69 (0.3)	184 (0.7)	309 (1.2)
No	30,884	87 (0.3)	182 (0.6)	330 (1.1)
Kidney function				
eGFR ≥30 ml/min/1.73 m^2^	46,285	32 (0.1)	90 (0.2)	178 (0.4)
eGFR <30 ml/min/1.73 m^2^	11,322	124 (1.1)	276 (2.4)	461 (4.1)
RAASi prescription^a^				
Yes	22,814	91 (0.4)	212 (0.9)	358 (1.6)
No	9209	58 (0.6)	131 (1.4)	233 (2.5)

a N = 32 023 (ODB-eligible: 1+ drug dispensed in ODB in 1-year prior to index date or age >65 yrs on index date).

With regard to other clinical outcomes within 7 calendar days of the hyperkalemia report, there were 155 (0.4%) cardiovascular deaths, 579 (1.0%) arrhythmias, 1782 (3.1%) cardiac resuscitations in the ED, 3609 (6.3%) hospitalizations, and 260 (0.5%) patients started dialysis.

### Sensitivity Analysis

The reanalysis of the full cohort to include cases where the potassium report was between a hospital or ED admission and discharge and include cases where there was a hospital or ED encounter prior to the report created a cohort of 58 857 individuals with a hyperkalemic result >6.2 mmol/L. There were 11 111 patients who had an ED encounter within 24 hours of the potassium result. The mean age was 65 (SD ± 18) years with 48% women. The mean potassium values were similar in the ED versus No-ED group (6.9 vs 6.8 mmol/L). Patients in the ED group had a higher Charlson Comorbidity Index (1.43 vs 0.73), lower eGFR (48 vs 68 mL/min/1.73 m^2^), higher use of loop diuretics (36.2% vs 27.2%), and higher use of potassium sparing diuretics (22.5% vs 15%).

All-cause mortality in the sensitivity analysis was similar to the main cohort. There were 971 (1.6%) deaths within 7 calendar days with 401 (0.7%) within 1 day and 656 (1.1%) within 3 days. Secondary outcomes were also similar in the sensitivity analysis. Within 7 days of the potassium report, there were 235 cardiovascular deaths (0.5%), 712 arrhythmias (1.2%), 2142 cardiac resuscitations in the ED (3.6%), 4439 (7.5%) hospitalizations, and 316 dialysis starts (0.5%).

Among all patients with a potassium result >6.5 mmol/L, there were a total of 349 deaths (1.4% of the population) within 7 days of the hyperkalemia report. Among these deaths, 94 (0.4% of the population) occurred within 1 calendar day of the report and 223 of these deaths (0.9% of the population) occurred within 3 calendar days of the report.

### Initial Potassium Results in the ED

In the study timeframe, 3316 patients lived in a catchment area where the hospital laboratory results were captured in OLIS. Among these patients, the mean outpatient potassium value was 6.8 mmol/L (SD ± 0.7). The mean initial potassium value within 24 hours of the ED registration was 5.3 mmol/L (SD ± 1.0). The distribution of the first potassium value in the ED is summarized in Table 4. When comparing the outpatient potassium result with the initial potassium result within 24 hours of an ED registration, the per-individual mean change was –1.5 mmol/L (SD ± 1.3).

**Table 4.** Distribution of Initial Potassium Values in the ED.

Initial potassium value in ED	Number of individuals (%) N = 3319
≤5.0 mmol/L	1290 (38.9)
5.1-5.5 mmol/L	607 (18.3)
5.6-6.0 mmol/L	622 (18.8)
6.1-6.5 mmol/L	444 (13.4)
6.5-7.0 mmol/L	228 (6.9)
>7.0 mmol/L	125 (3.8)

## Discussion

This large population-based study with over 71 000 hyperkalemia (>6.2 mmol/L) results and over 57 000 unique patients highlights that outpatient hyperkalemia is a common clinical scenario. Interestingly, despite the widely accepted concern that hyperkalemia requires urgent management, only 7469 (13.0%) of patients had an ED encounter within 24 hours of the hyperkalemia report. Patients with an ED encounter appeared to be sicker with higher baseline healthcare utilization, along with a higher comorbidity burden, and worse kidney disease. While both groups showed similar use of ACE inhibitors/ARBs, the ED group had higher prevalence of β-blockers, loop diuretics, and spironolactone, possibly due to their increased comorbidities. Finally, this study highlighted important clinical outcomes. Specifically, it showed that in the entire cohort, 1.2% of patients die within 7 days of an outpatient potassium result >6.2 mmol/L.

When patients presented to the ED with an outpatient serum potassium value of >6.2 mmol/L, the mean value of the potassium result within 24 hours of registration was only 5.3 mmol/L with a per-individual mean difference of –1.5 mmol/L. Furthermore, 2519 of the 3316 patients had a potassium value <6.0 mmol/L, suggesting that factitious hyperkalemia after an outpatient laboratory reports a value >6.2 mmol/L is common. It is important to recognize that there are slight differences in outpatient laboratories, which use serum values, and hospital laboratories, which use plasma values. As plasma potassium values tend to be about 0.5 mmol/L lower than serum values,^21^ the results cannot be directly compared. Previous studies demonstrate that results between the 2 specimen types correlate well, and that there is greater variability at higher potassium values.^22^ Nonetheless, this discrepancy between measured potassium levels underscores the complexity of determining which patients truly require further management. Additional research is warranted to create an accurate prediction tool for factitious hyperkalemia.

With over 71 000 individuals with a potassium >6.2 mmol/L, severe hyperkalemia emerges as a frequent concern in clinical practice. Despite this, only 7469 people in this cohort had an ED encounter within 24 hours. It is important to note that some patients may have been contacted to go to the ED, but did not agree to go or presented >24 hours later, which is not captured in this study. However, it is interesting, there was no significant difference in the potassium levels between patients who had an ED encounter versus those who did not. This suggests that factors beyond potassium levels, such as patient comorbidities and physician preferences, likely influenced the decision to refer patients to the ED. However, the frequency of ED encounters decreased (only 10%) with values >7.5 mmol/L. This counterintuitive decrease may be because such extreme values are often deemed incompatible with life, suggesting a high likelihood of analytical error, such as hemolysis or factitious hyperkalemia.

The incidence of ED encounters was most frequent for values between 6.7 and 7.4 mmol/L. Comparing potassium distributions between patients with an ED encounter and those without, patients were more likely to have an ED encounter within 24 hours when healthcare providers received direct notification of hyperkalemic results exceeding 6.6 mmol/L. This suggests that ED referrals may also be influenced by critical lab notifications. Previous guidelines have varied in defining thresholds for urgent ED intervention, ranging from >6.0 to >6.5 mmol/L, underscoring the need for standardizing outpatient hyperkalemia management protocols across different healthcare settings. Together, these data suggest the need for standardization of outpatient hyperkalemia values requiring urgent follow-up.

In the context of outcomes, within 7 days of an outpatient potassium result >6.2 mmol/L, there was a notable incidence of serious events, including a 1.2% chance of mortality, along with outcomes of arrhythmias, cardiac arrests, and hospitalizations. Subgroup analysis revealed the mortality was higher in patients with lower kidney function (eGFR <30 mL/min/1.73 m^2^) and patients not on RAASi. Observational studies show that outpatients who remain on RAASi, even after a hyperkalemic episode, have significantly lower all-cause and cardiovascular mortality than those who discontinue.^23,24^ This further suggests the survival benefit of RAASi compared with those not on these therapies. Alternatively, it is possible that patients on RAASi may have been able to be corrected more quickly by having an offending agent to discontinue. Regardless, these findings suggest the serious nature of hyperkalemia and the need for urgent management. This descriptive study cannot determine if an ED encounter mitigates the mortality risk. However, we are planning future studies to compare ED and No-ED groups to explore this question further. Nonetheless, this study reaffirms that hyperkalemia constitutes a life-threatening condition demanding timely intervention.

### Study Strengths and Limitations

This study has several strengths. This was a large population-based study that described a cohort of patients with hyperkalemia values >6.2 mmol/L designed to describe the difference between outpatients with hyperkalemia who presented to the ED compared with those who did not. As the data were collected from the diverse population of Ontario, Canada, this study is generalizable to similar populations and healthcare settings. In addition, the value of hyperkalemia that was used is consistent with current guidelines on when physicians are informed of urgent potassium levels.

This study also has some limitations. Many causes of death, including trauma, endocrine disorders, and malignancy, can lead to hyperkalemia and may confuse the association between hyperkalemia and mortality.^25-27^ In this study, it could not be determined if the patient death was due to the high potassium level or by another cause. Within ICES databases, sudden cardiac death is subject to misclassification or may not be reported for patients who died outside of an ED or hospital setting. In this study, the first ED potassium was created on the likely scenario that a blood test would be completed within 24 hours of an ED encounter. It is possible, though unlikely, that a patient would register for an ED encounter and have blood work done at an outside facility within 24 hours without any blood work done in the ED. It is also important to highlight that this study could not determine other management strategies of providers. For instance, rather than sending a patient to the ED, they may have asked the patient to alter their diet or change medications. Finally, the data for medication prescriptions were limited to only patients who were 65 years of age and older due to the limitations of coverage through the provincial drug plan. The use of sodium zirconium cyclosilicate and patiromer were unable to be assessed as they are not on the drug formulary during the study period.

## Conclusion

Outpatient hyperkalemia presents a substantial clinical challenge with serious potential outcomes. This study highlights significant variability in the management of hyperkalemia exceeding 6.2 mmol/L among healthcare providers. Notably, only 13% of patients had an ED encounter within 24 hours of receiving the potassium level. Given the observed 1% risk of mortality within a week of a potassium result >6.2 mmol/L, future research should focus on determining whether early ED care mitigates the risk. In addition, this study provides a real-world description of factitious hyperkalemia, where the per-individual mean change of potassium was –1.5 mmol/L. Clarifying the impact of ED management on patient outcomes and better predicting factitious hyperkalemia is crucial for optimizing care protocols yet balancing health resource utilization for outpatient hyperkalemia.

## Supplemental Material

sj-docx-1-cjk-10.1177_20543581251356568 – Supplemental material for Frequency, Management, and Outcomes of Outpatient Hyperkalemia: A Population-Based Cohort Study Supplemental material, sj-docx-1-cjk-10.1177_20543581251356568 for Frequency, Management, and Outcomes of Outpatient Hyperkalemia: A Population-Based Cohort Study by Michael Chiu, Nivethika Jeyakumar, Graham Smith, Danielle M. Nash, Mohamed Abou El Hassan, Dana Bailey, Peter Catomaris, Kika Veljkovic, Louise Moist, Amit X. Garg and Arsh K. Jain in Canadian Journal of Kidney Health and Disease

sj-docx-2-cjk-10.1177_20543581251356568 – Supplemental material for Frequency, Management, and Outcomes of Outpatient Hyperkalemia: A Population-Based Cohort Study Supplemental material, sj-docx-2-cjk-10.1177_20543581251356568 for Frequency, Management, and Outcomes of Outpatient Hyperkalemia: A Population-Based Cohort Study by Michael Chiu, Nivethika Jeyakumar, Graham Smith, Danielle M. Nash, Mohamed Abou El Hassan, Dana Bailey, Peter Catomaris, Kika Veljkovic, Louise Moist, Amit X. Garg and Arsh K. Jain in Canadian Journal of Kidney Health and Disease

sj-docx-3-cjk-10.1177_20543581251356568 – Supplemental material for Frequency, Management, and Outcomes of Outpatient Hyperkalemia: A Population-Based Cohort Study Supplemental material, sj-docx-3-cjk-10.1177_20543581251356568 for Frequency, Management, and Outcomes of Outpatient Hyperkalemia: A Population-Based Cohort Study by Michael Chiu, Nivethika Jeyakumar, Graham Smith, Danielle M. Nash, Mohamed Abou El Hassan, Dana Bailey, Peter Catomaris, Kika Veljkovic, Louise Moist, Amit X. Garg and Arsh K. Jain in Canadian Journal of Kidney Health and Disease
