# Supplementary material for: Frequency, Management, and Outcomes of Outpatient Hyperkalemia: A Population-Based Cohort Study
Source: Can J Kidney Health Dis. 2025 Jul 29;12:20543581251356568. doi: 10.1177/20543581251356568 (PMC12317165; doi:10.1177/20543581251356568)
Supplement: sj-docx-1-cjk-10.1177_20543581251356568 – Supplemental material for Frequency, Management, and Outcomes of Outpatient Hyperkalemia: A Population-Based Cohort Study [file sj-docx-1-cjk-10.1177_20543581251356568.docx]

Appendix A: Summary of Expert Panel Recommendations for Hyperkalemia Treatment

| **Expert Guidelines** | **Urgent potassium threshold to send patient to ED** |
| --- | --- |
| Canadian Cardiovascular Society 2016^11^ | >6.0 mmol/L |
| KDIGO 2020^12^ | >6.0 mmol/L or any ECG changes |
| Renal Association 2020^13^ | >6.5 mmol/L  OR  Acutely unwell patients if >5.5 mmol/L, particularly in presence of acute kidney injury |
| European Guidelines for Resuscitation 2021^14^ | >6.5 mmol/L |
|  |  |
| **Consensus Statements** | **Urgent potassium threshold to send patient to ED** |
| French Society of Cardiology^15^ | >6.0 mmol/L |
| Italian Society of Nephrology^16^ | >6.5 mmol/L – urgent intervention needed |
