# Supplementary material for: Frequency, Management, and Outcomes of Outpatient Hyperkalemia: A Population-Based Cohort Study
Source: Can J Kidney Health Dis. 2025 Jul 29;12:20543581251356568. doi: 10.1177/20543581251356568 (PMC12317165; doi:10.1177/20543581251356568)
Supplement: sj-docx-3-cjk-10.1177_20543581251356568 – Supplemental material for Frequency, Management, and Outcomes of Outpatient Hyperkalemia: A Population-Based Cohort Study [file sj-docx-3-cjk-10.1177_20543581251356568.docx]

**Table 2:** Baseline Characteristics of Patients with an Outpatient Hyperkalemic Result

|  | **Full Population** | **ED encounter** | **No-ED encounter** | **Std. Diff ^a^** |
| --- | --- | --- | --- | --- |
|  | **57,607** | **7469** | **50,138** |  |
| **Characteristic** | **N (%)** | **N (%)** | **N (%)** |  |
| *Demographics* |  |  |  |  |
| Age, years, Mean (SD) | 64.9 (18.2) | 69.8 (15.4) | 64.2 (18.4) | 33% |
| 18-<30 | 2731 (4.7) | 156 (2.1) | 2575 (5.1) | 16% |
| 30-<40 | 3638 (6.3) | 219 (2.9) | 3419 (6.8) | 18% |
| 40-<50 | 5272 (9.2) | 386 (5.2) | 4886 (9.7) | 17% |
| 50-<60 | 8464 (14.7) | 909 (12.2) | 7555 (15.1) | 8% |
| 60+ | 37,502 (65.1) | 5799 (77.6) | 31,703 (63.2) | 32% |
| Female | 27,503 (47.7) | 3389 (45.4) | 24,114 (48.1) | 5% |
| Rurality^b^ |  |  |  |  |
| Yes | 5081 (8.8) | 827 (11.1) | 4254 (8.5) | 9% |
| No | 52,435 (91.0) | 6625 (88.7) | 45,810 (91.4) | 9% |
| Missing | 91 (0.2) | 17 (0.2) | 74 (0.1) | 3% |
|  |  |  |  |  |
| *Health care utilization in the prior year* |  |  |  |  |
| Hospital visits, Mean (SD) | 0.6 (1.4) | 1.1 (1.9) | 0.6 (1.8) |  |
| Median (IQR) | 0 (0-1) | 0 (0-2) | 0 (0-1) |  |
| 0 | 39,226 (68.1) | 3940 (52.8) | 35,286 (70.4) | 37% |
| 1-5 | 17,673 (30.7) | 3314 (44.4) | 14,359 (28.6) | 33% |
| 6-10 | 634 (1.1) | 190 (2.5) | 444 (0.9) | 12% |
| 11+ | 74 (0.1) | 25 (0.3) | 49 (0.1) | 4% |
|  |  |  |  |  |
| ED visits, Mean (SD) | 1.0 (2.0) | 1.6 (2.7) | 0.9 (1.8) |  |
| Median (IQR) | 0 (0-1) | 1 (0-2) | 0 (0-1) |  |
| 0 | 34,338 (59.6) | 3229 (43.2) | 31,109 (62.0) | 38% |
| 1-5 | 21,622 (37.5) | 3797 (50.8) | 17,825 (35.6) | 31% |
| 6-10 | 1331 (2.3) | 357 (4.8) | 974 (1.9) | 16% |
| 11+ | 316 (0.5) | 86 (1.2) | 230 (0.5) | 8% |
|  |  |  |  |  |
| Family physician visits, Mean (SD) | 11.3 (12.7) | 13.0 (14.5) | 11.1 (12.4) |  |
| Median (IQR) | 8 (4-14) | 9 (5-16) | 8 (4-14) |  |
| 0 | 1732 (3.0) | 245 (3.3) | 1487 (3.0) | 2% |
| 1-5 | 19,042 (33.1) | 2035 (27.2) | 17,007 (33.9) | 5% |
| 6-10 | 15,428 (26.8) | 1999 (26.8) | 13,429 (26.8) | 0% |
| 11+ | 21,405 (37.2) | 3190 (42.7) | 18,215 (36.3) | 3% |
|  |  |  |  |  |
| Nephrologist visits, Mean (SD) | 0.7 (2.5) | 1.2 (3.5) | 0.6 (2.3) |  |
| Median (IQR) | 0 (0-0) | 0 (0-1) | 0 (0-0) |  |
| 0 | 46,692 (81.1) | 5314 (71.1) | 41,378 (82.5) | 7% |
| 1-5 | 9345 (16.2) | 1774 (23.8) | 7571 (15.1) | 22% |
| 6-10 | 1066 (1.9) | 232 (3.1) | 834 (1.7) | 9% |
| 11+ | 504 (0.9) | 149 (2.0) | 355 (0.7) | 11% |
|  |  |  |  |  |
| *Ordering Physician Characteristics* |  |  |  |  |
| Physician specialty ordering index OLIS test (type) | |  |  |  |
| Family physician | 43,221 (75.0) | 4975 (66.6) | 38,246 (76.3) | 22% |
| Emergency medicine | * | * | * |  |
| Cardiology | 1247 (2.2) | 235 (3.1) | 1012 (2.0) | 7% |
| Nephrology | 4623 (8.0) | 799 (10.7) | 3824 (7.6) | 11% |
| Internal medicine | 1931 (3.4) | 332 (4.4) | 1599 (3.2) | 6% |
| Other | 6585 (11.4) | 1128 (15.1) | 5457 (10.9) | 13% |
|  |  |  |  |  |
| Years in practice |  |  |  |  |
| Mean (SD) | 25.9 (11.5) | 23.9 (12.0) | 26.2 (11.5) | 20% |
| Median (IQR) | 26 (17-34) | 23 (14-33) | 26 (18-35) |  |
| <5 | 1109 (1.9) | 238 (3.2) | 871 (1.7) | 10% |
| 5-10 | 4790 (8.3) | 876 (11.7) | 3914 (7.8) | 13% |
| >10 | 47,175 (81.9) | 5812 (77.8) | 41,363 (82.5) | 12% |
| Missing | 4533 (7.9) | 543 (7.3) | 3990 (8.0) | 3% |
| Rural practice |  |  |  |  |
| Yes | 2915 (5.1) | 439 (5.9) | 2476 (4.9) | 4% |
| No | 50,112 (87.0) | 6479 (86.7) | 43,633 (87.0) | 1% |
| Missing | 4580 (8.0) | 551 (7.4) | 4029 (8.0) | 2% |
| Canadian graduate |  |  |  |  |
| Yes | 32,466 (56.4) | 4252 (56.9) | 28,214 (56.3) | 1% |
| No | 17,104 (29.7) | 1901 (25.5) | 15,203 (30.3) | 11% |
| Missing | 8037 (14.0) | 1316 (17.6) | 6721 (13.4) | 12% |
| *Comorbidities, %* |  |  |  |  |
| Charlson score, Mean (SD) | 0.84 (1.62) | 1.37 (1.95) | 0.76 (1.55) | 35% |
| Charlson score, Median (IQR) | 0 (0-1) | 0 (0-2) | 0 (0-1) |  |
| 0 | 40,483 (70.3) | 4089 (54.7) | 36,394 (72.6) | 38% |
| 1 | 4286 (7.4) | 672 (9.0) | 3614 (7.2) | 7% |
| 2 | 4813 (8.4) | 937 (12.5) | 3876 (7.7) | 16% |
| 3+ | 8025 (13.9) | 1771 (23.7) | 6254 (12.5) | 29% |
|  |  |  |  |  |
| Diabetes | 26,723 (46.4) | 4360 (58.4) | 22,363 (44.6) | 28% |
| Congestive Heart Failure | 10,886 (18.9) | 2108 (28.2) | 8778 (17.5) | 26% |
| Hypertension | 38,470 (66.8) | 5893 (78.9) | 32,577 (65.0) | 31% |
| Kidney transplant | 234 (0.4) | 52 (0.7) | 182 (0.4) | 4% |
|  |  |  | | |
| *Laboratory Measurements in the prior year* | | | | |
| Index potassium value, Mean (SD) | 6.8 (0.78) | 6.8 (0.65) | 6.8 (0.80) | 3% |
|  |  |  |  |  |
| eGFR (ml/min/1.73m^2^), Mean (SD) | 65.0 (33.5) | 47.6 (30.2) | 67.6 (33.2) | 63% |
| ≥ 60, % | 30,863 (53.6) | 2241 (30.0) | 28,622 (57.1) | 57% |
| 45 - 59, % | 6873 (11.9) | 1048 (14.0) | 5825 (11.6) | 07% |
| 30 - 44, % | 8549 (14.8) | 1532 (20.5) | 7017 (14.0) | 17% |
| < 30, % | 11,322 (19.7) | 2648 (35.5) | 8674 (17.3) | 42% |
|  |  |  |  |  |
| Urine ACR (mg/mmol) |  |  |  |  |
| Mean (SD) | 48.63 (117) | 67.61 (138) | 45.1 (113) | 18% |
| Median (IQR) | 4 (1-30) | 10 (2-64) | 3 (1-25) |  |
| < 3 | 32,711 (56.8) | 1335 (17.9) | 10,247 (20.4) | 6% |
| 3-30 | 11,582 (20.1) | 1222 (16.4) | 5869 (11.7) | 14% |
| > 30 | 7091 (12.3) | 1348 (18.0) | 4875 (9.7) | 24% |
| Missing | 6223 (10.8) | 3564 (47.7) | 29,147 (58.1) | 21% |
| *Medication use in the prior 4 months, for those eligible for ODB, %* | | | | |
| ODB eligible | 32,023 (55.6) | 5078 (68.0) | 26,945 (53.7) | 30% |
| ACE inhibitor | 12,837 (40.1) | 2118 (41.7) | 10,719 (39.8) | 4% |
| Angiotensin receptor blocker | 9547 (29.8) | 1533 (30.2) | 8014 (29.7) | 1% |
| Antibiotics | 10,818 (33.8) | 1920 (37.8) | 8898 (33.0) | 10% |
| Aliskiren | 178 (0.6) | 25 (0.5) | 153 (0.6) | 1% |
| Beta blockers | 13,681 (42.7) | 2438 (48.0) | 11,243 (41.7) | 13% |
| Calcineurin inhibitors | 219 (0.7) | 49 (1.0) | 170 (0.6) | 4% |
| Loop diuretics | 9272 (29.0) | 1759 (34.6) | 7513 (27.9) | 14% |
| Potassium sparing diuretics | 5253 (16.4) | 1081 (21.3) | 4172 (15.5) | 15% |
| Thiazide diuretics | 3866 (12.1) | 659 (13.0) | 3207 (11.9) | 3% |
| NSAIDs | 3891 (12.2) | 525 (10.3) | 3366 (12.5) | 7% |
| Potassium supplements | 750 (2.3) | 111 (2.2) | 639 (2.4) | 1% |
| SGLT2 inhibitors | 1227 (3.8) | 220 (4.3) | 1007 (3.7) | 3% |
| Spironolactone | 4692 (14.7) | 988 (19.5) | 3704 (13.7) | 16% |
|  |  |  |  |  |

*SD = standard deviation; IQR = interquartile range; ODB = Ontario Drug Benefit; OLIS = Ontario Laboratory Reporting System; eGFR = estimated glomerular filtration rate; ACR = albumin to creatinine ratio; ACE = angiotensin converting enzyme; NSAID = non-steroidal inflammatory drug; SGLT2 = sodium glucose transporter 2*

*^a^ Standardized difference was used to compare hyperkalemic patients who presented to an ED vs. hyperkalemic patients who did not present to an ED. Standardized differences are less sensitive to sample size than traditional hypothesis tests. They provide a measure of difference between groups with respect to a pooled standard deviation. A standardized difference ≥1% is considered a meaningful difference between groups*

*^b^ Rural status was defined as residence within a community <10,000 persons*

** Due to a small cell, this value was collapsed with the Other group*
